# Clinicopathological and prognostic significance of homeobox transcript antisense RNA expression in various cancers: A meta-analysis

**DOI:** 10.1097/MD.0000000000007084

**Published:** 2017-06-08

**Authors:** Sai-Nan Min, Tai Wei, Xiang-Ting Wang, Li-Ling Wu, Guang-Yan Yu

**Affiliations:** a Department of Oral and Maxillofacial Surgery, Peking University School and Hospital of Stomatology, National Engineering Laboratory for Digital and Material Technology of Stomatology, and Beijing Key Laboratory of Digital Stomatology, Beijing; b School of Life Science, University of Science and Technology of China, Hefei; c Department of Physiology and Pathophysiology, Peking University School of Basic Medical Sciences, Key Laboratory of Molecular Cardiovascular Sciences, Ministry of Education, and Beijing Key Laboratory of Cardiovascular Receptors Research, Beijing, China.

**Keywords:** cancer, clinicopathological feature, HOTAIR, prognosis

## Abstract

**Background:**

Increased expression of the homeobox (HOX) transcript antisense RNA (HOTAIR) has been reported in multiple types of malignancies and enhances the proliferation and migration of cancer cells. However, the association between HOTAIR expression and tumor progression and prognosis remains controversial. We performed a meta-analysis to clarify the association between the expression of HOTAIR and the clinicopathological features and prognosis in different cancers.

**Methods:**

A systematic search of the PubMed, Web of Science, EMBASE, and Ovid databases was conducted, up to September 2016, for eligible studies involving HOTAIR expression and malignancies. The odds ratios (ORs), hazard ratios (HRs), and corresponding 95% confidence intervals (CIs) were calculated using fixed- or random-effect models. Any publication bias was evaluated using Begg and Egger tests, and adjusted using the trim and fill method if a bias existed.

**Results:**

A total of 4116 patients from 44 studies were included in our meta-analysis. The results showed that high HOTAIR expression was associated with an advanced clinical tumor stage (OR = 3.90, 95% CI = 3.02–5.03, *P* < .001), lymph node metastasis (OR = 3.11, 95% CI = 2.15–4.49, *P* < .001), poor differentiation of the tumor (OR = 1.56, 95% CI = 1.01–2.41, *P* = .03), and worse prognosis (HR = 2.16, 95% CI = 1.73–2.69, *P* < .001) in different cancer types. HOTAIR expression was more predictive in monitoring the clinical tumor stage of patients and there was no significant heterogeneity or publication bias found in the analysis.

**Conclusion:**

Our meta-analysis suggests that HOTAIR is positively correlated with tumor development and negatively correlated with clinical outcome. Thus, an increase in HOTAIR expression may be a potential biomarker for tumor progression and evaluation of prognosis.

## 1 Introduction

Cancer is still considered to be a major challenge for modern medicine.^[1]^ Tumor specific markers can be useful for monitoring and evaluating tumor progression and therapy efficiency. However, there is still a shortage of highly sensitive and specific markers for assessing cancer progression and prognosis.^[2]^ Long noncoding RNAs (lncRNAs) are noncoding transcripts longer than 200 nucleotides that have complicated biological functions at the epigenetic, transcriptional, and posttranscriptional levels.^[3]^ Several studies have demonstrated that lncRNAs can act as oncogenes or tumor suppressors, and are aberrantly expressed in a number of cancers, making lncRNAs a hot topic in the field of translational medicine.^[4–9]^ Among them, the homeobox (HOX) transcript antisense RNA (HOTAIR) is one of the most studied lncRNAs in oncology.

HOTAIR, also known as HOXAS, HOXC-AS4, and HOXC11-AS1, is a 2158 nucleotide antisense transcript derived from the HOXC gene cluster on chromosome 12q13.13.^[10]^ It is the first lncRNA found to regulate gene expression in trans.^[10,11]^ The best-known function for HOTAIR is as a scaffold molecule. The 5′ sequence of HOTAIR binds to the polycomb repressive complex 2, while the 3′ sequence binds to the lysine specific demethylase 1 complex. Thus, HOTAIR ties several epigenetic regulators together and recruits them to specific genes promoter/loci,^[11]^ resulting in histone H3 lysine27 tri-methylation and histone H3 lysine4 demethylation, consequently silencing target genes.^[12,13]^ Recently, high expression of HOTAIR has been observed in multiple types of malignancies including gastric, hepatocellular, and lung cancers and has been associated with tumor progression and prognosis.^[14–16]^ For instance, HOTAIR expression is elevated in gastric cancer tissues and patients with high HOTAIR expression have a shorter overall survival (OS) compared with patients with lower expression levels of HOTAIR.^[14]^ Elevated HOTAIR expression is also detected in hepatocellular cancer tissues and is strongly correlated with an advanced clinical stage.^[15]^ In nonsmall cell lung cancer, increased HOTAIR expression is correlated with an increase in lymph node metastasis.^[16]^ However, the association between elevated HOTAIR expression and lymph node metastasis has not been identified in patients with colorectal cancer.^[17]^ Additionally, a number of studies show that upregulation of HOTAIR contributes to the proliferation and invasion of cancer cells.^[14–16]^ Taken together, these studies indicate that HOTAIR might be a key regulator in the carcinogenesis and progression of different types of cancers.

Although several meta-analyses have investigated the relationship between HOTAIR and cancer,^[18–26]^ the results of previous studies are inconsistent especially with regard to the clinicopathological features. Moreover, previous studies are limited by sample size or mainly focus on the prognostic value of HOTAIR. Therefore, we conducted a meta-analysis to systematically explore the clinicopathological and prognostic value of HOTAIR in different types of cancers.

## 2 Materials and methods

### 2.1 Search strategy

This study was performed according to the standard protocols for meta-analysis. Two investigators (Min and Wei) independently searched electronic databases including PubMed, Web of Science, EMBASE, and Ovid for original studies in English using the Medical Subject Heading terms: (“HOX transcript antisense RNA” or “HOTAIR” or “HOXAS” or “HOXC-AS4” or “HOXC11-AS1”) and (“carcinoma” or “cancer” or “neoplasm” or “malignancy” or “tumor”). The acquired articles, published up to September 2016, were screened according to the title, abstract, and study content. In addition, a manual search of the reference lists of the relevant studies was performed in order to include studies that might have been omitted by the original search. Since our analyses were based on previously published studies, no ethical approval was required.

### 2.2 Inclusion and exclusion criteria

Studies were included if they met the following criteria: articles were published in English as full-text research manuscripts; HOTAIR expression was examined in cancer tissues and the relationship between HOTAIR levels and clinicopathological parameters and/or cancer prognosis was evaluated; odds ratios (ORs) for investigating clinicopathological parameters and hazard ratio (HR) for estimating prognostic outcome were provided or there was enough information to extract them; and if the same research team reported data from overlapping patient populations in different publications, only the largest dataset was chosen.

Reviews, case reports, letters, conference abstracts, animal experiments, and non-English language articles were excluded. Articles that provided insufficient information on estimating ORs or cancer prognosis were also excluded.

### 2.3 Data extraction

Two authors (Min and Wei) extracted the following information from the studies: the last name of the first author, year of publication, geographic region, tumor type, sample size, assay methods, cut-off values, clinicopathological and prognostic parameters, and other relevant data. Any disagreement was resolved by discussion until the authors reached a consensus.

### 2.4 Quality assessment of included studies

The Newcastle-Ottawa scale rating system was used to assess the quality of the studies included in our meta-analysis, ranging from 0 to 9 stars.^[27]^ Studies with more than 6 stars were considered to be high quality studies.^[28,29]^ Two authors performed the assessment independently and any disagreement was resolved by discussion.

### 2.5 Statistical analysis

The ORs with corresponding 95% confidence intervals (CIs) were used to assess the correlation between HOTAIR expression and the clinicopathological parameters in patients with cancers. The clinicopathological parameters included tumor size T1 and T2 versus T3 and T4; the absence or presence of lymph node metastasis; clinical stage I–II versus III–IV; well differentiation versus moderate–poor differentiation; age less than 60 years old versus age equal to or more than 60 years old; and male versus female.

The HRs with corresponding 95% CIs were used to estimate the association between HOTAIR expression and cancer prognosis. Data were directly extracted from the original articles if the authors had provided the exact HRs and 95% CIs. For studies that did not provide the HRs or 95% CIs, we estimated the values using the available information such as the number of events, patients at risk, and *P* values. Some studies presented the prognosis results as Kaplan–Meier curves and in such cases the curves were read by the Engauge Digitizer (version 4.0) to construct HR estimates based on the method described by Tierney et al.^[30]^ HRs and corresponding 95% CIs were transformed to their natural logarithms to stabilize the variance and normalize the distribution.^[31]^

The Chi-Square test was used to assess the heterogeneity of the studies included and the significance was set at *P* < .05. The Higgins *I*^2^ statistic was used to estimate heterogeneity and when *I*^2^ ≤ 50% the fixed model was used, otherwise the random-effect model was applied and subgroup analysis was used to determine the potential cause of the heterogeneity. Funnel plots were used to detect any potential publication bias combined with the Begg and Egger linear regression tests. Sensitivity analysis was employed to identify the influence of individual studies on the combined effect values. All statistical analyses were performed using the Stata 13.0 software (Stata Corporation, College Station, TX). All *P* values were two-sided.

## 3 Results

### 3.1 Study selection and characteristics

**Figure 1:**
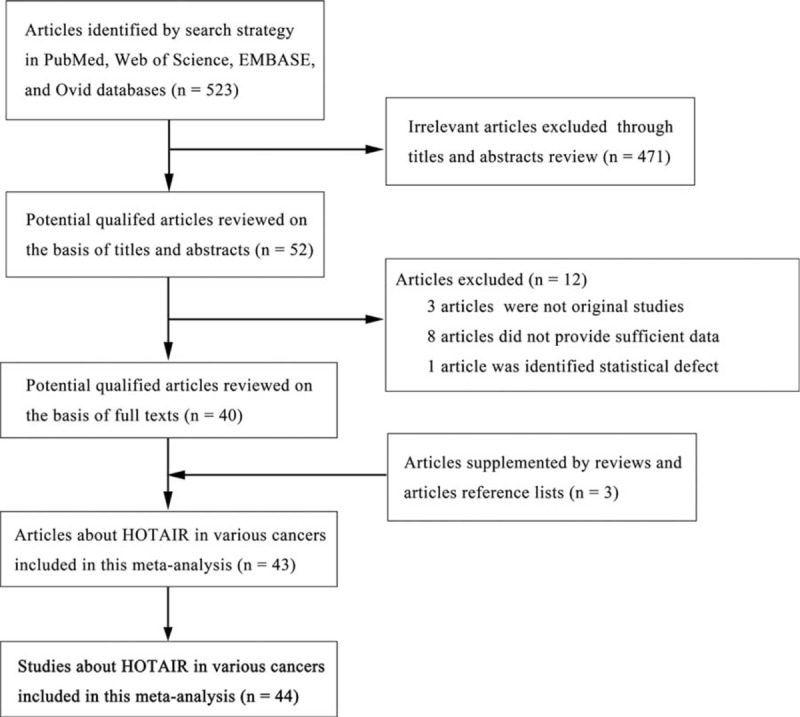
Flow diagram of study selection procedure.

The flow diagram (Fig. 1) shows that a total of 523 articles were retrieved using our search strategy. We excluded 471 articles because they were found to contain irrelevant or duplicate information following a detailed review of the titles and abstracts. Further evaluation of the remaining 52 papers revealed that 8 articles did not contain sufficient data and 3 articles were not original studies, and were eliminated from our analysis. In addition, 1 article was excluded because of a statistical defect. However, an additional 3 articles were included after screening reference lists. As a result, there were 43 eligible articles^[14–17,32–70]^ that contained 44 studies because 1 article analyzed 2 different cancer subtypes.^[14]^

The detailed characteristics of the 44 studies included in our meta-analysis were summarized in Table 1. The articles were published worldwide with 37 articles from Asian countries and 7 articles from Western countries. The number of cases ranged from 30 to 336 and included 23 different types of cancer, including gastric cancer, breast cancer, oral squamous cell carcinoma (OSCC), nonsmall cell lung cancer, hepatocellular cancer, and bladder cancer (Table 1). The cancers included in this meta-analysis were divided into further groups based on their organ of origin: estrogen-dependent carcinomas (n = 11), digestive system carcinomas (n = 21), respiratory system carcinomas (n = 4), OSCCs (n = 2), and others (n = 6). Thirty-nine studies performed quantitative real-time PCR (qRT-PCR) to detect HOTAIR expression and 4 studies used RNA in-situ hybridization (ISH). One study analyzed the prognostic value of HOTAIR by microarray. Of the clinicopathological variables, age, gender, clinical tumor stage, lymph node metastasis, degree of differentiation, and tumor size were selected, and their relationships with HOTAIR expression were analyzed. The number of studies utilized in our meta-analysis varied depending on the specific clinicopathological feature or prognosis. In the total 44 studies, the clinical tumor stage was evaluated in 20 studies, information on lymph node metastasis was provided in 23 studies, tumor differentiation was investigated in 19 studies, tumor size was examined in 26 studies, and 32 studies evaluated the prognostic significance of HOTAIR expression.

**Table 1:**
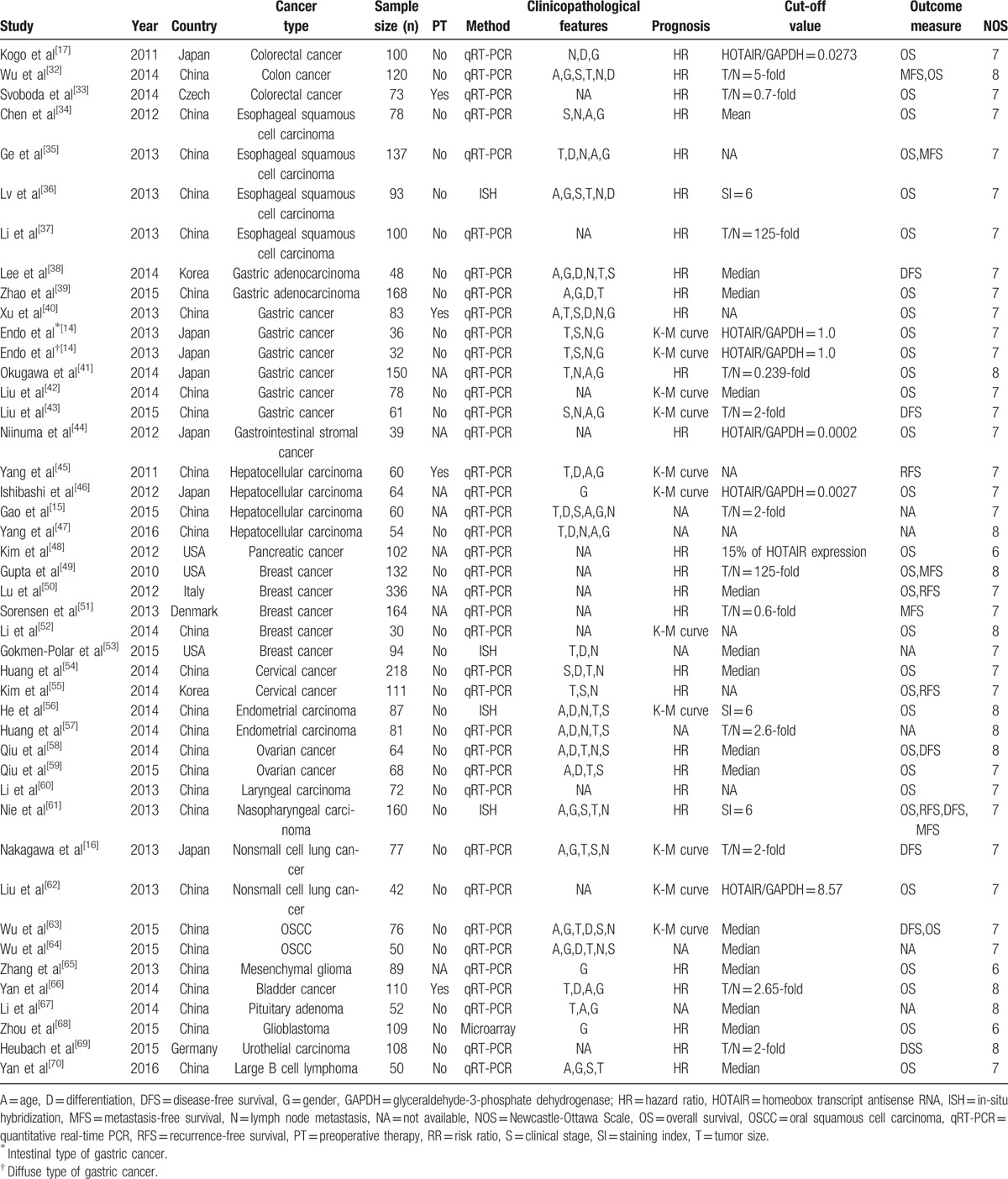
Main characteristics of eligible studies.

### 3.2 Study quality

The qualities of the eligible papers were assessed using Newcastle-Ottawa scale. The scores of these studies ranged from 6 to 8. Therefore, all eligible articles were taken into account.

### 3.3 HOTAIR expression and clinicopathological characteristics in various cancers

In order to explore the relationship between HOTAIR expression and various clinicopathological parameters, OR values and corresponding CIs were pooled, respectively, within different variables (Table 2). There was no significant correlation between HOTAIR expression and age (OR = 0.95, 95% CI = 0.79–1.15, *P* = .69) or gender (OR = 1.09, 95% CI = 0.90–1.33, *P* = .36).

**Table 2:**
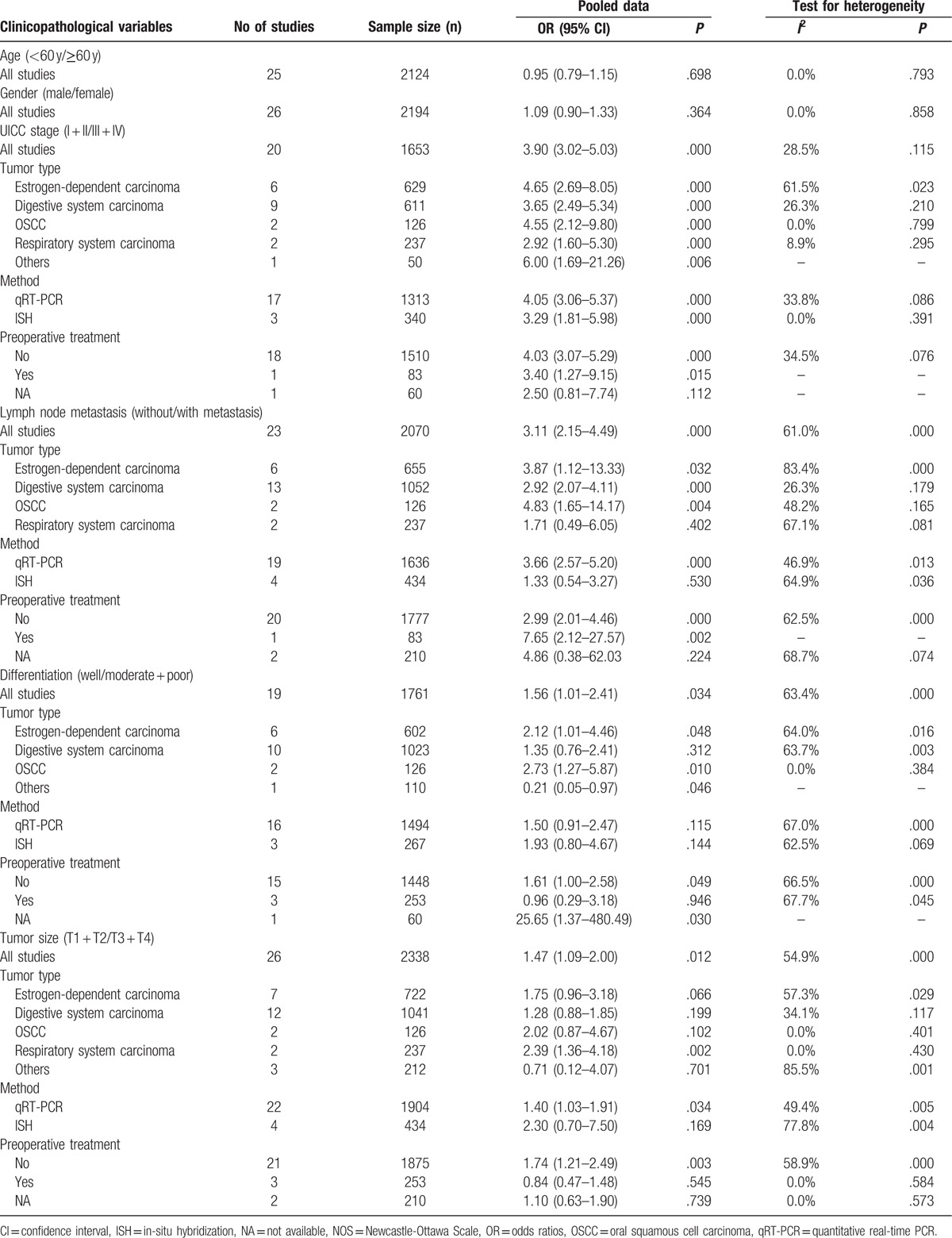
Results of subgroup analysis of pooled ORs with regard to clinicopathological variables.

#### 3.3.1 HOTAIR and clinical tumor stage

**Figure 2:**
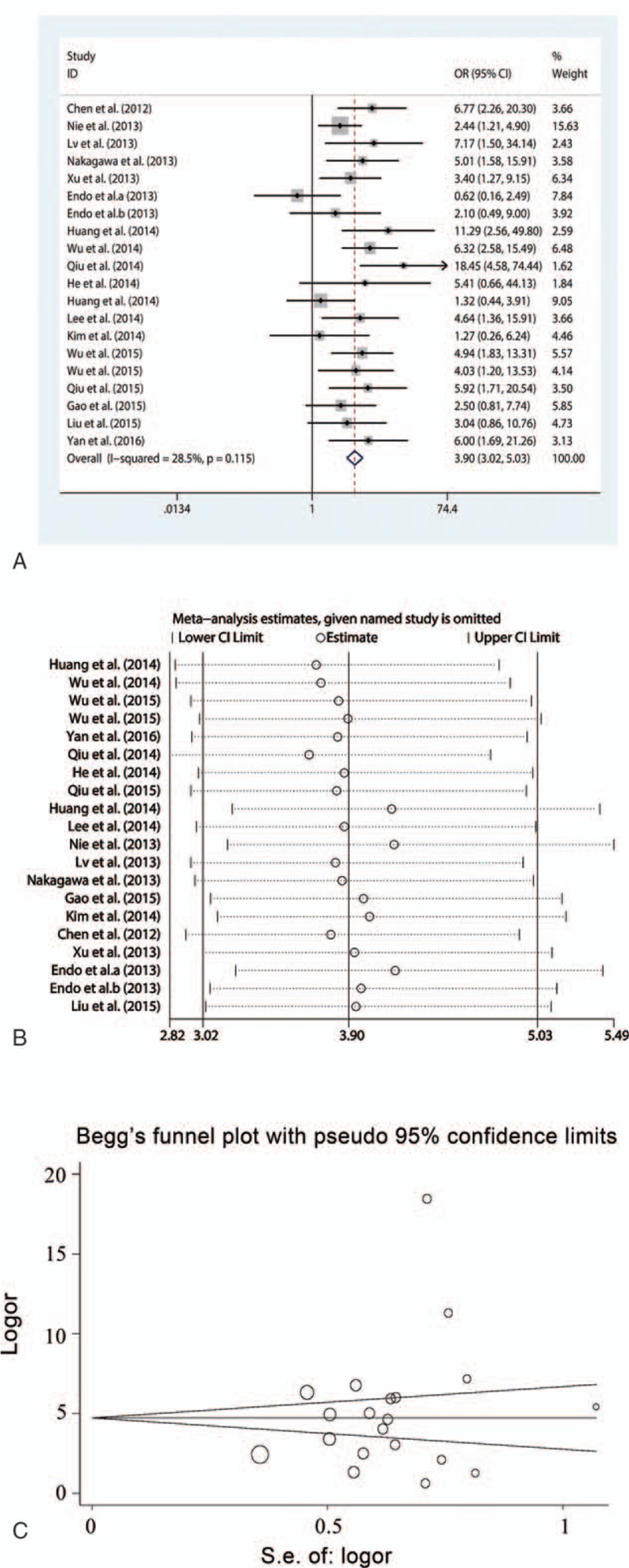
Meta-analysis of the pooled ORs of HOTAIR with clinical stages of cancer patients. (A) Forest plot of the pooled ORs. (B) Sensitivity analysis. (C) Begg funnel plot for publication bias. HOTAIR = homeobox transcript antisense RNA, OR = odds ratio.

A total of 20 studies involving 1653 patients were included in the analysis between HOTAIR expression and clinical tumor stage. A fixed-effect model was applied because of the lower interstudy heterogeneity (*I*^2^ = 28.5%, *P* = .11). The results showed that HOTAIR expression significantly correlated with clinical tumor stage (OR = 3.90, 95% CI = 3.02–5.03, *P* < .001), indicating that the clinical tumor stage was more advanced in patients with high HOTAIR expression compared with patients with low HOTAIR expression (Fig. 2A). Subgroup analysis was performed to assess the association between HOTAIR and the clinical tumor stage of patients based on cancer type, detection method, and preoperative treatment. HOTAIR expression was associated with clinical tumor stage in all cancer types assessed in our meta-analysis including estrogen-dependent carcinomas (OR = 4.65, 95% CI = 2.69–8.05, *P* < .001), digestive system carcinomas (OR = 3.65, 95% CI = 2.49–5.34, *P* < .001), respiratory system carcinomas (OR = 2.92, 95% CI = 1.60–5.30, *P* < .001), OSCCs (OR = 4.55, 95% CI = 2.12–9.80, *P* < .001), and other carcinomas (OR = 6.00, 95% CI = 1.69–21.26, *P* = .006). The association between HOTAIR expression and clinical tumor stage could be detected in patients by qRT-PCR and ISH, suggesting that HOTAIR has the potential to be a stable predictor to evaluate cancer progression. Preoperative treatment did not affect the results. Sensitivity analysis showed that the OR estimates of the relevant data altered between the lower and upper CI limits, suggesting that the corresponding data and our conclusions were stable and reliable (Fig. 2B). A publication bias was not detected using the Begg (*P* = .31) and Egger funnel plots (*P* = .24) (Fig. 2C).

#### 3.3.2 HOTAIR and lymph node metastasis

**Figure 3:**
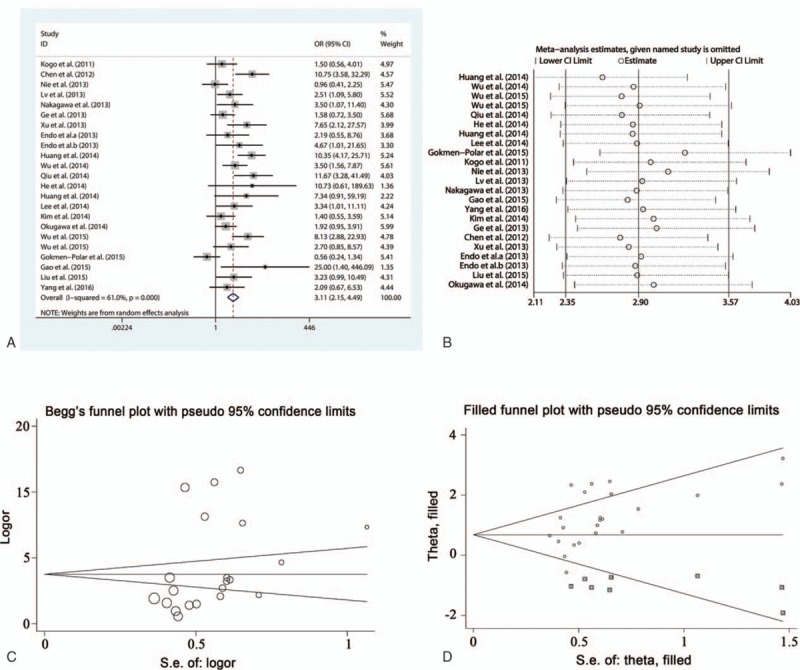
Meta-analysis of the pooled ORs of HOTAIR with lymph node metastasis of cancer patients. (A) Forest plot of the pooled ORs. (B) Sensitivity analysis. (C) Begg funnel plot for publication bias. (D) Filled funnel plot using “trim-and-fill” method. Circles: included studies; diamonds: presumed missing studies. HOTAIR = homeobox transcript antisense RNA, OR = odds ratio.

A total of 2070 patients in 23 studies were analyzed to determine an association between HOTAIR expression and lymph node metastasis. Since a moderate heterogeneity (*I*^2^ = 61.0%, *P* < .001) existed across the studies, the random effect model was used in this analysis. The result showed that HOTAIR expression was significantly associated with lymph node metastasis (OR = 3.11, 95% CI = 2.15–4.49, *P* < .001), suggesting that patients with increased HOTAIR expression were at high risk of developing lymph node metastases (Fig. 3A). Subgroup analyses showed that high HOTAIR expression was closely correlated with lymph node metastasis in estrogen-dependent carcinomas (OR = 3.87, 95% CI = 1.12–13.33, *P* = .03), digestive system carcinomas (OR = 2.92, 95% CI = 2.07–4.11, *P* < .001), and OSCCs (OR = 4.83, 95% CI = 1.65–14.17, *P* = .004), but not in respiratory system carcinomas (OR = 1.71, 95% CI = 0.49–6.05, *P* = .40). Heterogeneity did not exist in the subgroup of patients with digestive system carcinomas (*I*^2^ = 26.3%, *P* = .17) and OSCCs (*I*^2^ = 48.2%, *P* = .16). However, the correlation between HOTAIR expression and lymph node metastasis was only observed in the patients tested by qRT-PCR (OR = 3.66, 95% CI = 2.57–5.20, *P* < .001), and not by ISH. Moreover, HOTAIR expression strongly related to lymph node metastasis in patients with preoperative treatment (OR = 7.65, 95% CI = 2.12–27.57, *P* = .002) and without preoperative treatment (OR = 2.99, 95% CI = 2.01–4.46, *P* < .001). Sensitivity analysis demonstrated that the OR estimates were not influenced by excluding single article successively (Fig. 3B). Although Egger funnel plot was symmetric (*P* = .090), Begg test showed a significant publication bias (*P* = .01) (Fig. 3C). Hence, the trim and fill method was adopted. After incorporating 8 assumptive studies, the statistical significance between HOTAIR expression and lymph node metastasis still existed (OR = 1.982, 95% CI = 1.317–2.981, *P* = .001), showing that the results were reliable (Fig. 3D).

#### 3.3.3 HOTAIR and tumor differentiation

**Figure 4:**
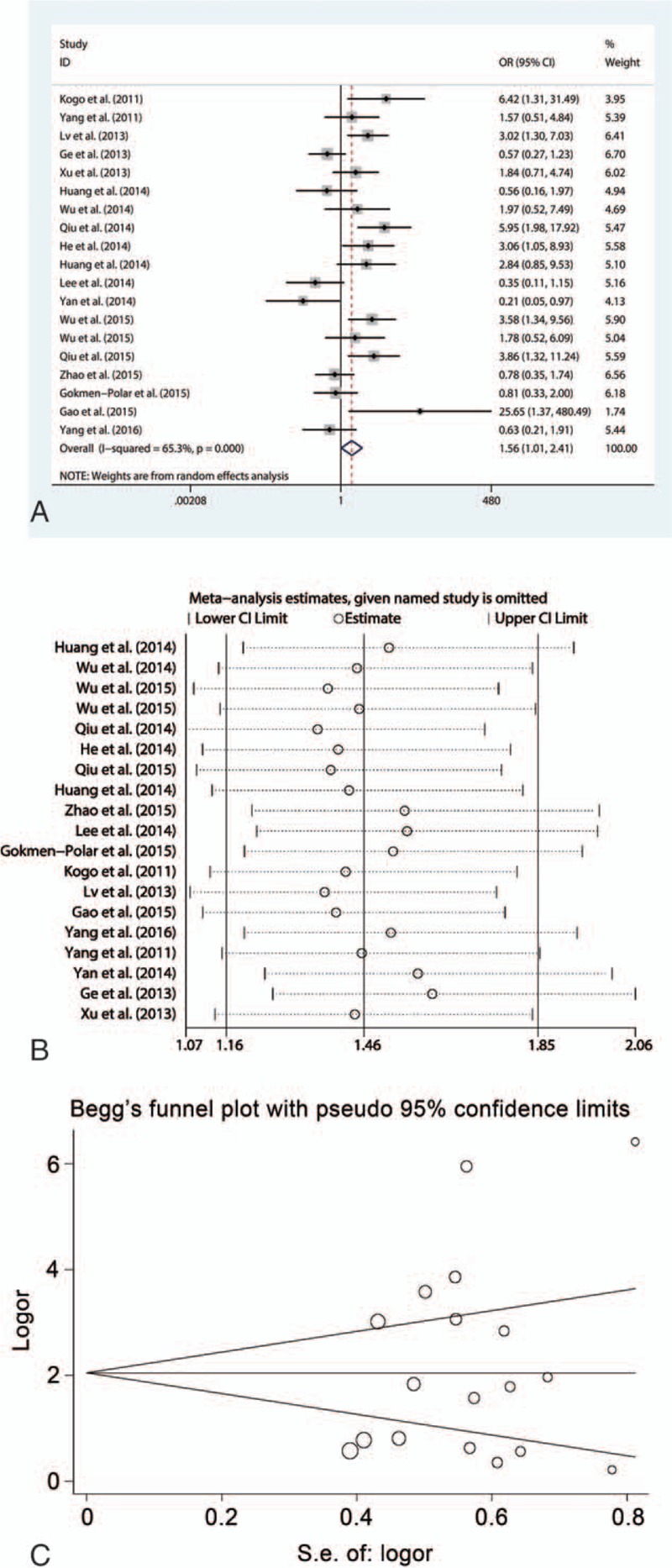
Meta-analysis of the pooled ORs of HOTAIR with differentiation of cancer patients. (A) Forest plot of the pooled ORs. (B) Sensitivity analysis. (C) Begg funnel plot for publication bias. HOTAIR = homeobox transcript antisense RNA, OR = odds ratio.

To investigate the association between HOTAIR expression and tumor differentiation, a total of 19 studies involving 1761 patients were analyzed. A random effect model was applied owing to the moderate heterogeneity (*I*^2^ = 63.4%, *P* < .001). The results demonstrated that high HOTAIR expression significantly correlated with poor differentiation (OR = 1.56, 95% CI = 1.01–2.41, *P* = .034), suggesting that increased HOTAIR expression might be an indicator of pathological grade (Fig. 4A). Subgroup analysis showed that the relationship between HOTAIR expression and differentiation was significant in estrogen-dependent carcinomas (OR = 2.12, 95% CI = 1.01–4.46, *P* = .04) with a moderate heterogeneity (*I*^2^ = 64.0%, *P* = .01) and OSCCs (OR = 2.73, 95% CI = 1.27–5.87, *P* = .010; *I*^2^ = 0.0%, *P* = .38) without any heterogeneity, but not in digestive system carcinomas (OR = 1.35, 95% CI = 0.76–2.41, *P* = .31) or other carcinomas (OR = 0.21, 95% CI = 0.05–0.97, *P* = .04). In addition, statistical significance only existed in patients who had not received any preoperative therapy (OR = 1.61, 95% CI = 1.00–2.58, *P* = .04). Sensitivity analysis showed that all estimates were within the upper and lower CI limits, suggesting that our data were reliable (Fig. 4B). No publication bias was detected by the Begg (*P* = .36) and Egger test (*P* = .294) (Fig. 4C).

#### 3.3.4 HOTAIR and tumor size

**Figure 5:**
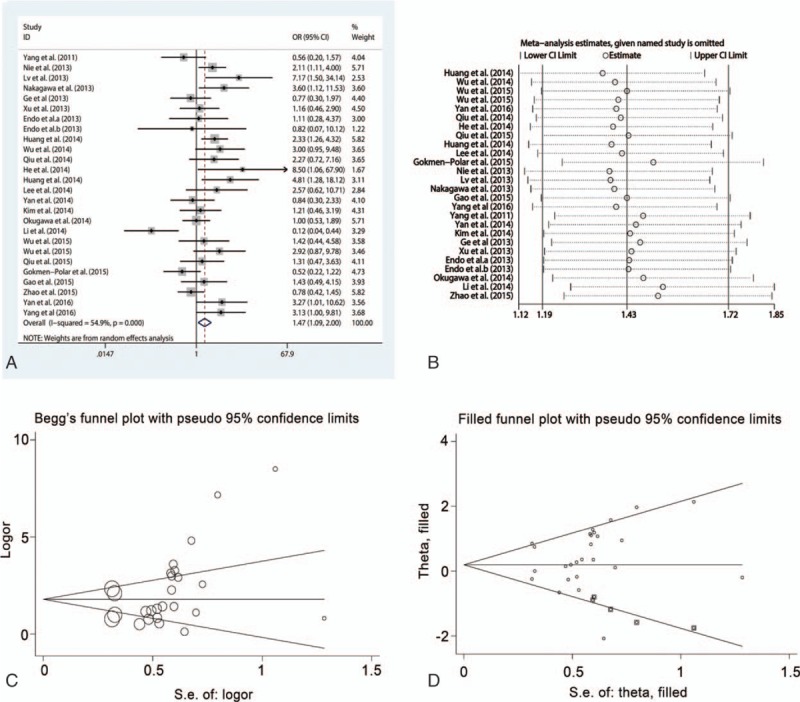
Meta-analysis of the pooled ORs of HOTAIR with tumor size of cancer patients. (A) Forest plot of the pooled ORs. (B) Sensitivity analysis. (C) Begg funnel plot for publication bias. (D) Filled funnel plot using “trim-and-fill” method. HOTAIR = homeobox transcript antisense RNA, OR = odds ratio.

The association between HOTAIR expression and tumor size was analyzed in 26 studies that included 2338 patients. There was a moderate heterogeneity (*I*^2^ = 54.9%, *P* < .001) and the random effect model was adopted. As Fig. 5A shows, high HOTAIR expression was significantly correlated with large tumor size (OR = 1.47, 95% CI = 1.09–2.00, *P* = .01). Subgroup analysis showed that the statistical significance was found in respiratory system carcinomas (OR = 2.39, 95% CI = 1.36–4.18, *P* = .002) without heterogeneity (*I*^2^ = 0.0%, *P* = .43), but not in estrogen-dependent carcinomas, digestive system carcinomas, and OSCCs. The significant correlation between HOTAIR expression and tumor size was identified by qRT-PCR (OR = 1.40, 95% CI = 1.03–1.91, *P* = .03), but not by ISH. In addition, the correlation was identified in patients who had not received preoperative treatment (OR = 1.74, 95% CI = 1.21–2.49, *P* = .003). Sensitivity analysis was performed and showed that the estimates of ORs varied between the CI limits, indicating that the results were stable (Fig. 5B). However, a significant publication bias was identified using the Begg (*P* = .02) and Egger funnel plots (*P* = .003) with visible asymmetry (Fig. 5C). The trim and fill method was performed by supplementing hypothetical studies for further analysis. However, the statistical significance between HOTAIR expression and tumor size disappeared after incorporating the hypothetical studies using the random effect model (OR = 1.212, 95% CI = 0.889–1.651, *P* = .22) (Fig. 5D). Therefore, these conclusions should be used cautiously.

### 3.4 HOTAIR expression and prognosis in various cancers

**Figure 6:**
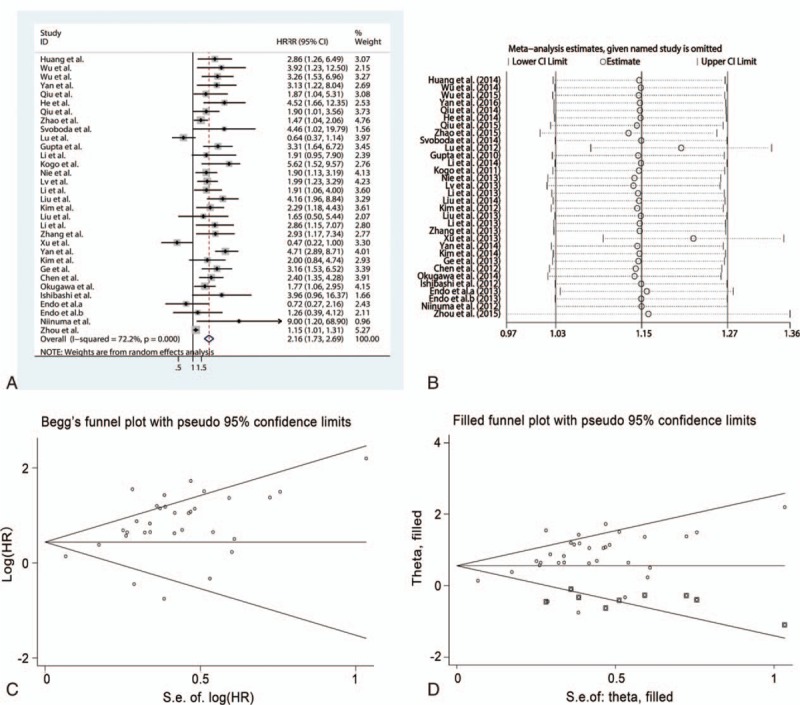
Meta-analysis of the pooled HRs of HOTAIR with prognosis of cancer patients. (A) Forest plot of the pooled HRs. (B) Sensitivity analysis. (C) Begg funnel plot for publication bias. (D) Filled funnel plot using “trim-and-fill” method. HOTAIR = homeobox transcript antisense RNA, HR = hazard ratio.

A total of 3207 patients from 32 studies were included to explore the prognostic value of HOTAIR expression in human cancers. The random-effect model was used to combine HRs and corresponding 95% CIs owing to the significant heterogeneity (*I*^2^ = 72.2%, *P* < .001). The results revealed that elevated HOTAIR expression was associated with poor prognosis in cancer patients (HR = 2.16, 95% CI = 1.73–2.69, *P* < .001) (Fig. 6A). Subgroup analysis showed that HOTAIR expression was associated with OS of patients in all types of cancers, such as estrogen-dependent carcinomas (HR = 2.00, 95% CI = 1.25–3.21, *P* = .004), digestive system carcinomas (HR = 2.11, 95% CI = 1.56–2.85, *P* < .001), OSCCs (HR = 3.26, 95% CI = 1.53–6.95, *P* = .002), and respiratory system carcinomas (HR = 2.04, 95% CI = 1.34–3.11, *P* = .001) (Table 3). A significant association existed between HOTAIR expression and prognosis using three detection methods. The correlation existed in patients without preoperative treatment (HR = 2.22, 95% CI = 1.76–2.80, *P* < .001), but not in patients who received preoperative treatment. Sensitivity analysis showed that the result was stable according to estimates of the ORs (Fig. 6B). A publication bias was detected by the Egger test (*P* < .001), but not by the Begg test (*P* = .39) (Fig. 6C). Therefore, the trim and fill method was performed and showed that the recalculated HR did not change significantly (HR = 1.746, 95% CI = 1.426–2.138, *P* < .001) for OS (Fig. 6D).

**Table 3:**
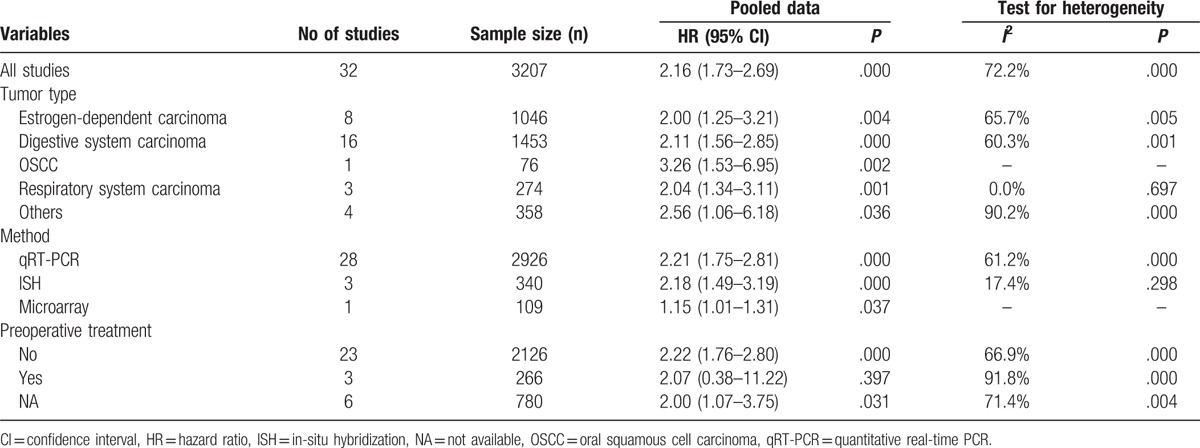
Results of subgroup analysis of pooled HRs with regard to overall survival.

## 4 Discussion

By integrating 44 studies with 4116 patients, our comprehensive meta-analysis revealed that a higher expression of HOTAIR was correlated with an advanced clinical stage, lymph node metastasis, poor differentiation, and a worse prognosis. In addition, the association between HOTAIR and tumor size was identified with a publication bias. These results suggested that increased HOTAIR expression might be used as a promising biomarker for determining tumor progression and prognosis.

Several meta-analyses have investigated the correlation between HOTAIR expression and clinicopathological features.^[18–21]^ Elevated HOTAIR expression is reported to be associated with advanced clinical stage and lymph node metastasis in gastric cancer from a meta-analysis involving 10 studies.^[18]^ However, the relationship between HOTAIR expression and clinical stage is not identified when the data on hepatocellular and gastric cancers are pooled in 4 studies.^[20]^ The clinicopathological value of HOTAIR expression is controversial, and most studies have focused primarily on malignancies of the digestive system. Various cancer types and limited study sizes may contribute to the discrepancy in results. We performed a comprehensive meta-analysis to investigate the clinicopathological value of high HOTAIR expression in various types of cancers. The results showed that high HOTAIR expression was significantly correlated with advanced clinical tumor stage, lymph node metastasis, poor differentiation, and large tumor size. Notably, the pooled OR value for clinical tumor stage was higher than the pooled OR values for other clinicopathological characteristics and no significant heterogeneity and publication bias was found, indicating that HOTAIR expression might be useful in monitoring the clinical stage of cancers. Moreover, preoperative treatment did not alter the results on clinical tumor stage and lymph node metastasis, but did influence the predictive effect of HOTAIR expression on differentiation and tumor size.

To further analyze the role of HOTAIR in different cancers, subgroup analysis was performed based on the type of cancer. For clinical tumor stage, high levels of HOTAIR were associated with advanced clinical stage in all cancer types in this study. For lymph node metastasis, the high level of HOTAIR expression might be more meaningful in predicting lymph node metastasis in estrogen-dependent carcinomas, digestive system cancers, and OSCCs. However, both relationships were only observed in cancers of the digestive system in previous analyses.^[18,20,21]^ We found that high HOTAIR expression was related to poor differentiation in estrogen-dependent cancers and OSCCs, although the correlation was not identified in previous studies.^[18–21]^ Our results showed that HOTAIR expression correlated with a larger tumor size, though the statistical significance was only found in cancers of the respiratory system and there was a publication bias, suggesting that large-scale investigations were required. There are several explanations for the discrepancy in predictive role of HOTAIR expression in various types of cancers. First, the effects of HOTAIR expression may vary in a cancer-specific manner. For example, the dysregulation of estrogen plays a key role in the tumorigenesis of breast cancer and HOTAIR expression can be induced by estrogen exposure.^[71]^ Human papillomavirus infection is a risk factor of several cancers such as tongue and cervical cancers.^[72,73]^ Human papillomavirus type 16 oncoprotein E7 promotes HOTAIR expression and upregulates metastasis-related genes by competing with polycomb repressive complex 2 for HOTAIR binding.^[73]^ Second, there are limited studies on OSCCs and respiratory system cancers, suggesting that future larger studies are required for these cancer types.

Several meta-analyses have showed that HOTAIR expression negatively correlates with prognosis in various types of cancer.^[20–26]^ However, unchanged levels of HOTAIR expression have also been reported in breast cancer.^[24]^ In order to evaluate the prognostic value of HOTAIR in different cancers, we included 32 studies in our meta-analysis. Our results showed that patients with higher HOTAIR expression had a shorter OS than those with a lower HOTAIR expression, which is consistent with previous studies. Subgroup analysis uncovered a relationship between high HOTAIR expression and poor prognosis in all cancer types studied. Chemotherapy is a common clinical therapy for cancers, and many studies have reported that HOTAIR plays a crucial role in drug resistance. Upregulation of HOTAIR is found in cisplatin-resistant gastric cancer cells, and it promotes cell proliferation by inhibiting microRNA (miRNA)-126 expression and activating the PI3K/AKT/MRP1 pathway.^[74]^ Furthermore, HOTAIR has been shown to enhance the resistance of ovarian cancer cells to cisplatin via the wnt/β-catenin pathway.^[75]^ The involvement of HOTAIR in drug resistance further confirms its prognostic value. However, our subgroup analysis showed that a significant association between HOTAIR and prognosis was only observed in patients that did not undergo preoperative treatment, suggesting that preoperative chemotherapy might affect the regulation of HOTAIR.

Although there is a positive correlation between the expression of HOTAIR and tumor progression, the underlying mechanism of HOTAIR in tumorigenesis and development remains unclear. The high expression of HOTAIR promotes OSCC cells metastases by recruiting the enhancer of zeste homolog 2 and repressing E-cadherin.^[63]^ HOTAIR upregulates human epithelial growth factor receptor 2 expression, thereby enhancing proliferation and migration of gastric cancer cells by sponging miR-331–3p.^[42]^ In addition, HOTAIR enhances migration and invasion of breast cancer cells by inhibiting miR-7 expression.^[76]^ Several factors might affect HOTAIR expression in cancers. For example, the methylation status of the HOTAIR downstream intergenic CpG island is positively correlated with HOTAIR expression in breast cancer.^[77]^ Moreover, type I collagen, which is enriched in the tumor microenvironment, is reported to promote HOTAIR expression via Myc in lung cancer cells.^[78]^ The expression of HOTAIR can also be induced by estrogen through estrogen response elements of the HOTAIR promoter.^[79]^ These factors might contribute to the aberrant abundance of HOTAIR in tumor tissues.

miRNAs are small noncoding RNAs containing about 21 to 23 nucleotides, which posttranscriptionally suppress gene expression. Previous studies demonstrate that miRNAs are also potential biomarkers in cancers.^[80,81]^ Recently, the posttranscriptional interaction between HOTAIR and miRNA has been confirmed by a number of studies. HOTAIR enhances hepatocellular cancer cell proliferation and tumorigenicity in vivo by suppressing miR-218, which acts as tumor suppressor in bladder cancer.^[80,82]^ In gallbladder cancer, HOTAIR promotes cell proliferation and metastasis by inhibiting miR-130a expression.^[83]^ The cross-regulation between HOTAIR and miRNAs in cancers reveals a novel strategy for using a combination of HOTAIR and miRNAs for tumor progression and prognostic markers. A study demonstrated that the combination of HOTAIR and miR-21 is more accurate for screening laryngeal cancer using the receiver-operating characteristic curve.^[84]^ However, owing to insufficient evidence, more clinical data are required for further evaluation.

Our study has some limitations. First, the definitions regarding cut-off values varied between the studies, which likely contributed to some of the heterogeneity. Thus, consensus cut-off values are required in future investigations. Second, HR estimates and 95% CIs were extracted from Kaplan–Meier curves in several studies that did not provide accurate values. The HR estimates and 95% CIs obtained in this manner may not be accurate. Last, a publication bias was observed between HOTAIR expression and tumor size even though the trim and fill method was performed. This indicates that the relationship between HOTAIR expression and tumor size should be used cautiously.

In summary, our meta-analysis demonstrates that high HOTAIR expression may serve as a potential indicator for advanced clinical tumor stage, lymph node metastasis, poor differentiation, and worse prognosis. HOTAIR might be a more prospective predictor of clinical tumor stage in cancer patients. However, more studies are required to verify the mechanism of HOTAIR in tumor development and its usage as a prognostic marker.
